# *Giardia duodenalis* and Its Secreted PPIB Trigger Inflammasome Activation and Pyroptosis in Macrophages through TLR4-Induced ROS Signaling and A20-Mediated NLRP3 Deubiquitination

**DOI:** 10.3390/cells10123425

**Published:** 2021-12-06

**Authors:** Lin Liu, Yongwu Yang, Rui Fang, Weining Zhu, Jingxue Wu, Xiaoyun Li, Jay V. Patankar, Wei Li

**Affiliations:** 1 Heilongjiang Provincial Key Laboratory of Zoonosis, College of Veterinary Medicine, Northeast Agricultural University, Harbin 150030, China; liulinneau@163.com (L.L.); yyw230512@163.com (Y.Y.); fangrui7372@163.com (R.F.); jipinzhu1207@126.com (W.Z.); wujingxue00@163.com (J.W.); lixiaoyun@neau.edu.cn (X.L.); 2 Department of Medicine 1, University of Erlangen-Nuremberg, 91052 Erlangen, Germany; Jay.Patankar@uk-erlangen.de

**Keywords:** *Giardia duodenalis*, macrophage pyroptosis, TLR4, NLRP3 deubiquitination, A20, PPIB

## Abstract

The extracellular protozoan parasite *Giardia duodenalis* is a well-known and important causative agent of diarrhea on a global scale. Macrophage pyroptosis has been recognized as an important innate immune effector mechanism against intracellular pathogens. Yet, the effects of noninvasive *Giardia* infection on macrophage pyroptosis and the associated molecular triggers and regulators remain poorly defined. Here we initially observed that NLRP3 inflammasome-mediated pyroptosis was activated in *Giardia*-treated macrophages, and inhibition of ROS, NLRP3, or caspase-1 could block GSDMD cleavage, IL-1β, IL-18 and LDH release, and the cell viability reduction. We also confirmed that *Giardia*-induced NLRP3 inflammasome activation was involved in its K63 deubiquitination. Thus, six candidate deubiquitinases were screened, among which A20 was identified as an effective regulator. We then screened TLRs on macrophage membranes and found that upon stimulation TLR4 was tightly correlated to ROS enhancement, A20-mediated NLRP3 deubiquitination, and pyroptotic signaling. In addition, several *Giardia*-secreted proteins were predicted as trigger factors via secretome analysis, of which peptidyl-prolyl cis-trans isomerase B (PPIB) independently induced macrophage pyroptosis. This was similar to the findings from the trophozoite treatment, and also led to the TLR4-mediated activation of NLRP3 through K63 deubiquitination by A20. Collectively, the results of this study have significant implications for expanding our understanding of host defense mechanisms after infection with *G. duodenalis.*

## 1. Introduction

The intestinal protozoan parasite *Giardia duodenalis* is responsible for 280 million infections annually around the world [1]. *Giardia* infections remain a major public health burden in developing countries, and giardiasis has been included in WHO’s Neglected Disease Initiative [2]. The life cycle of *Giardia* includes two stages: the infectious cyst and disease-causing trophozoites. Susceptible hosts ingest cysts via contaminated food and water, then trophozoites are released from the cysts upon stimulation by gastric acid and bile and interact with and adhere to intestinal epithelial cells (IECs) via the ventral disc [3]. *G. duodenalis* poses a serious risk of infection in immunocompromised individuals and children; the infection often causes diarrhea, nausea, vomiting, and weight loss, and may also trigger latent inflammatory bowel disease and irritable bowel syndrome [4,5].

Combined innate and adaptive immune responses are needed to control *Giardia* infection and replication [6]. *Giardia* excretory–secretory products and variant-specific surface proteins (VSPs) have been known to cause high levels of serum and salivary IgA in infected patients [7,8,9]. Dendritic cells (DCs), mast cells, IECs, and macrophages have been shown to contribute to host defense responses in *Giardia* infection [6]. Mature DCs can produce proinflammatory cytokines including tumor necrosis factor (TNF)-α, interleukin (IL)-6, and IL-12 when stimulated by *Giardia* binding immunoglobulin protein [10]. Our recent studies have indicated that the apoptotic program can be initiated in IECs upon *Giardia* trophozoite treatment [11,12]. It has been shown that macrophages are involved in the immune response to *Giardia* infection via the activation of AKT/MAPK signaling [13]. Mouse macrophages can release extracellular traps to capture and kill *Giardia* [14]. However, despite these advancements, whether and how macrophages mediate host defense against noninvasive *Giardia* infection are still poorly understood.

Pyroptosis, a form of programmed cell death mediated by inflammatory caspases, involves both tissue homeostasis and an immune response [15]. Macrophage pyroptosis participates in direct parasite killing inside the host cells by causing inflammation [16]. Gasdermin D (GSDMD), a common executor of pyroptosis, can be cleaved by inflammasome-activated caspase (CASP)-1 into the N- and C-termini; N-GSDMD then oligomerizes and forms pores in the plasma membrane and increases membrane permeability, leading to pyroptosis and IL-1β and IL-18 release [17,18]. Inflammasomes are formed when pathogen-associated molecular patterns (PAMPs) are sensed by the AIM2- and NOD-like receptors (e.g., NLRP1a, NLRP3, and NLRC4) in the cytoplasm [19,20]. PAMPs can also be recognized by membrane-associated innate immune sensors, such as toll-like receptors (TLRs) 2 and 4 [21,22]. An elevated inflammatory response mediated by TLR2/MAPK signaling plays a role in giardiasis severity [13]. TLR4 is a vital mediator of the proinflammatory response via regulating multiple signaling pathways including NLRP3 inflammasome signaling [23]. The VSPs of *Giardia* were reported to activate TLR2 and TLR4 in HEK293 cells [24]. The latest research has shown that *Giardia* extracellular vesicles (EVs) activate NLRP3 inflammasome signaling via TLR2 [25]. Growing evidence shows that activation of the NLRP3 inflammasome involves its deubiquitination or phosphorylation [26,27]. It is, therefore, worthwhile to examine the correlation between noninvasive *Giardia* infection, TLR-mediated recognition of parasite PAMPs, NLRP3 deubiquitination, and the activation and regulation of inflammasomes and pyroptosis.

The functions of *Giardia*-secreted proteins have attracted increasing attention over the past years. Cysteine proteases secreted by *Giardia* can disrupt IEC junctional complexes and degrade chemokines, causing damage to the small intestine as noted in [28]. *Giardia*-secreted giardipain-1 is a likely contributor to the barrier damage and IEC apoptosis [29]. Three *Giardia* proteases instigate the macrophage-mediated inflammatory response via cleavage of the NF-ĸB p65 subunit [30]. Via secretome analysis, several proteins were found to be highly expressed in the culture supernatant of *G. duodenalis* assemblage A [31,32], such as pyridoxamine 5-phosphate oxidase (PNPO), peptidyl-prolyl cis-trans isomerase B (PPIB), and three tenascins. The potential function of these proteins in *Giardia*–host cell interactions needs further in-depth investigation. To date, there has been no report on the involvement of macrophage pyroptosis in *Giardia* infection. Here we provide the first evidence that *G. duodenalis* and its secreted PPIB can induce macrophage pyroptosis, and the associated trigger factors and regulators are explored.

## 2. Materials and Methods

### 2.1. Experimental Animals

Four-week-old C57BL/6 mice were housed under a 12 h light/dark cycle in temperature-controlled conditions. Water and food were available ad libitum throughout.

### 2.2. Cell Culture

The murine macrophage cell line J774A.1 was purchased from the Cell Bank of the Chinese Academy of Sciences (Shanghai, China). Cells were maintained in Dulbecco’s Modified Eagle’s medium (DMEM; Hyclone, Logan, UT, USA) containing 10% fetal bovine serum (FBS; Cellmax, Beijing, China) in a humidified 37 °C, 5% CO_2_ incubator. Peritoneal macrophages (PMs) were elicited from C57BL/6 mice following the intraperitoneal injection of 3% thioglycollate broth. Three days after injection, mice were euthanized and the PMs were harvested by peritoneal lavage with 5 mL DMEM. Erythrocytes were removed using ACK lysis buffer (Leagene, Beijing, China), and then, the remaining cells were cultured in DMEM supplemented with 10% FBS and 1% penicillin/streptomycin (Beyotime, Shanghai, China) and allowed to adhere for 24 h before treatment.

### 2.3. Parasite Culture

The *G. duodenalis* isolate WB (assemblage A) was used in this study (ATCC30957, Manassas, VA, USA). *Giardia* trophozoites were grown in filter-sterilized modified TYI-S-33 culture medium at 37 °C [33]. Unattached/dead parasites were removed from culture tubes by changing the medium. After cooling in an ice bath, the parasites were collected, counted by a hemocytometer, diluted to the designated amount using cell culture medium, and used to treat macrophages at a ratio of 10 parasites/cell.

### 2.4. Lactate Dehydrogenase (LDH) Assay

J774A.1 cells or PMs were inoculated into 96-well plates at a density of 3 × 10^4^ cells/well in a complete medium and incubated for 12 h. After 3 h of culture in DMEM without FBS, macrophages were treated with trophozoites for the indicated time periods, and those treated with 0.1% Triton X-100 were set as a positive control. LDH release was evaluated using an LDH Assay Kit according to the instruction manual (Beyotime, Shanghai, China). Absorbance was read at 490 nm using an enzyme-linked immunosorbent assay (ELISA) reader (BioTek, Winooski, VT, USA).

### 2.5. Annexin V/Propidium Iodide (PI) Assay

J774A.1 cells or PMs (5 × 10^5^ cells/well) were cultured in 12-well plates and treated with parasites for the indicated time periods. Cells were double-stained with Annexin V-FITC/PI (Beyotime, Shanghai, China) at room temperature (RT) in the dark for 20 min, and tested with flow cytometry using BD FACS Canto II (BD Biosciences, San Jose, CA, USA). The percentage of Annexin V-positive cells was quantified with BD FACSDiva software and results were processed by FlowJo software (Tree Star, Ashland, OR, USA).

### 2.6. Cell Viability Assays

J774A.1 cells or PMs were plated at a seeding density of 2 × 10^4^ cells/well in 96-well plates in complete medium and incubated for 12 h, the medium was then replaced with serum-free medium and incubated for 3 h. Cells were stimulated with parasites for the indicated time periods, and negative control wells containing just DMEM and trophozoites in DMEM were included. Cell viability was detected by a cell counting kit-8 (CCK-8; Apexbio, Houston, TX, USA) assay. Absorbance was read at a wavelength of 450 nm.

### 2.7. Transmission Electron Microscopy (TEM) Detection

PMs were treated with trophozoites for the indicated time periods. Treated macrophages were collected by trypsin digestion and fixed at RT overnight in phosphate buffer solution (PBS) including 2.5% glutaraldehyde. Fixed cells were dehydrated serially in ethanol (five steps from 50% to 100%) and embedded in Epon 812. Ultrathin sections (55-nm thick) were cut using a Power Tome XL ultramicrotome (Leica, Buffalo Grove, IL, USA), and then stained with 2% uranyl acetate and lead citrate. TEM images were taken using a Hitachi H-7650 electron microscope (Tokyo, Japan).

### 2.8. Reactive Oxygen Species (ROS) Detection

The intracellular ROS level was measured using 2′,7′-dichlorofluorescin diacetate (DCFH-DA) which is converted to fluorescent 2′,7′-dichlorofluorescin (DCF) in the presence of peroxides. J774A.1 cells or PMs (3 × 10^5^ cells/well) were pretreated with 10 μM DCFH-DA (Beyotime, Shanghai, China) at 37 °C for 20 min, washed with PBS, and treated with parasites for the indicated time periods. The DCF epifluorescence intensity was examined with a Lionheart FX Automated Microscope (BioTek, Winooski, VT, USA).

### 2.9. Detection of the Mitochondrial Membrane Potential (MMP)

Mitochondrial membrane integrity was assessed by JC-1 staining (Solarbio, Beijing, China). In undamaged mitochondria, the aggregated dye emits red fluorescence, whereas in cells with diminished MMP, the dye emits bright green fluorescence. MMP was evaluated by analyzing the ratio of red to green fluorescence. J774A.1 cells or PMs were treated with trophozoites for the indicated time periods. After treatment, cells were digested with trypsin, collected by centrifugation, resuspended with JC-1 staining solution, and incubated for 20 min at 37 °C. The fluorescence intensity was measured by flow cytometry and the results were analyzed with FlowJo software.

### 2.10. Quantitative Real-Time PCR (qPCR) Analysis

PMs were treated with trophozoites for the indicated time periods. Total RNA was extracted from cells using Trizol reagent (Invitrogen, Carlsbad, CA, USA). The cDNA was synthesized by reverse transcription from 1 μg of total RNA using the Hiscript 1st Strand cDNA Synthesis Kit (Vazyme, Nanjing, China). Amplification was carried out on an LC480 Lightcycler system (Roche, Indianapolis, IN, USA) using the SYBR Green qPCR Master Mix (Bimake, Houston, TX, USA), and β-actin was applied as an endogenous control. The specific qPCR primers used here can be found in Table S1. The relative mRNA expression in the control was taken by us as 1, and fold changes relative to control were shown. The relative expression of mRNA was quantified by the 2^−ΔΔCt^ method.

### 2.11. Western Blot Analysis

J774A.1 cells or PMs were treated with parasites for the indicated time periods. Total cellular proteins were extracted using RIPA lysis buffer (Beyotime, Shanghai, China) supplemented with 1% PMSF (Beyotime, Shanghai, China). The protein concentration was quantified using the enhanced BCA Protein Assay Kit (Beyotime, Shanghai, China). Proteins were separated by sodium dodecyl sulfate-polyacrylamide gel electrophoresis (SDS-PAGE), and transferred onto polyvinylidene fluoride membranes. The membranes were blocked with 5% skim milk for 1.5 h and probed with primary antibodies against IL-18, pro-IL-1β, mature-IL-1β, β-tubulin, NLRP3, pro-CASP1, cleaved CASP1, GSDMD, lysine 63 (K63)-linkage specific polyubiquitin, A20, TLR2, TLR4, and TLR5 at a dilution of 1:1000 at 4 °C overnight. The primary antibodies were obtained from three commercial sources (ABclonal, Wuhan, China; ABMART, Shanghai, China; Wanleibio, Shenyang, China). Blots were probed with a 1:5000 dilution of horseradish peroxidase-conjugated goat anti-rabbit secondary antibody (ABMART, Shanghai, China) and visualized by chemiluminescence detection (Syngene, Cambridge, UK). The signal intensity of the protein band was analyzed by NIH Image J software (Bethesda, MD, USA).

### 2.12. Immunofluorescence Assays

J774A.1 cells or PMs were stimulated with trophozoites for the indicated time periods, fixed in 4% paraformaldehyde for 30 min, and permeabilized with 0.25% Triton-X 100 for 10 min at RT. Nonspecific sites were blocked by incubation with Fc receptor blocking solution (Abace, Beijing, China) containing 2% normal rabbit serum (Bioss, Beijing, China) for 30 min. Cells were incubated with primary antibodies against apoptosis-associated speck-like protein containing a CARD (ASC) and TLR4 (1:100 each; ABclonal, Wuhan, China) at 4 °C overnight and subsequently with FITC-AffiniPure goat anti-rabbit IgG (H + L) (1:200; Jackson, West Grove, PA, USA) at 37 °C for 1 h protected from light. Cell nuclei were labeled by DAPI (2 μg/mL; Alphabio, Tianjin, China). Epifluorescence imaging was performed using the Lionheart FX Automated Microscope.

### 2.13. Flow Cytometry for TLR Detection

PMs were treated with parasites for the indicated time periods, harvested by trypsinization, fixed in 2% paraformaldehyde for 10 min, and blocked with Fc receptor blocking solution containing 10% normal rabbit serum for 15 min at RT. All samples were incubated with anti-TLR2/TLR4/TLR5 antibody (1:100 each) for 30 min and then treated with FITC-AffiniPure goat anti-rabbit IgG (H + L) (1:200) for 40 min at RT. Fluorescently-labeled cells were analyzed on a BD FACS Canto II running FACSDiva and FlowJo.

### 2.14. Co-Immunoprecipitation (Co-IP) Analysis

We performed co-IP for analysis of NLRP3 ubiquitination. J774A.1 cells or PMs were treated with trophozoites for the indicated time periods. Total cellular proteins were extracted using the lysis buffer mentioned earlier and incubated with protein A/G magnetic beads (Bimake, Houston, TX, USA) and anti-NLRP3 antibody (1:200) at 4 °C overnight. The immunoprecipitates were collected using a magnetic rack, and the sediments were resuspended in lysis buffer and boiled for 8 min. The eluted proteins were analyzed by Western blotting with the anti-NLRP3 antibody and the antibody recognizing K63-linked polyubiquitin chains (both diluted 1:1000 in PBS for use).

### 2.15. RNA Interference

Knockdown of A20 in PMs was performed using anti-A20 small interfering RNA (si A20; 5’-GGGUAGGUUUGAAGACUUAtt-3’). We acquired siA20 and the nontargeting control siRNA (scrambled siRNA; siNC) from TsingKe Biological Technology (Beijing, China). Several groups were included: untreated, lipofectamine6000-treated (lipo6000; Beyotime, Shanghai, China), *Giardia*-treated; lipo6000 + *Giardia*-treated, siNC-treated, siNC + *Giardia*-treated, siA20-treated, and siA20 + *Giardia*-treated. PMs were transfected at 70% confluence using siRNA at a concentration of 50 nM and lipo6000. In brief, siRNA and lipo6000 were diluted separately in OPTI-MEM medium (Gibco, Carlsbad, CA, USA), and then mixed at a 1:1 ratio and incubated for 5 min. At 48 h after siRNA transfection, qPCR and immunoblotting assays were performed to detect the silencing efficiency. Successfully transfected cells were treated with trophozoites for 12 h for further analysis.

### 2.16. Prokaryotic Expression of Giardia-Secreted Proteins

Among *Giardia*-secreted proteins highly expressed in the trophozoite culture supernatant as described before [31], PNPO (GL50803_5810), PPIB (GL50803_17163), and tenascin-1/2/3 (GL50803_114815/10330/16833) were randomly chosen and expressed in a prokaryotic expression system. The genes encoding these proteins were amplified using the primer sets shown in Table S2, and then cloned into the pCold I vector (TaKaRa, Ohtsu, Japan). The resulting plasmids pCold I-PNPO/PPIB/tenascins were transformed into *E. coli* strain BL21 (DE3) cells. Protein expression was induced with 1 mM isopropyl β-d-thiogalactoside (Solarbio, Beijing, China) at 16 °C for 20 h. The harvested cells were lysed using an ultrasonic cell crusher (XO-650D; Xianou, Nanjing, China). Precipitated proteins and supernatants were subjected to SDS-PAGE analysis. Before treatment, the recombinant proteins were purified with a HisTrap HP nickel column (SMART, Changzhou, China). We then used an endotoxin ELISA kit (Meimian Biotech, Yancheng, China) to measure if the purified proteins were contaminated with endotoxins that may be responsible for the observed activation. If contaminated, proteins were further purified with Endotoxin Removal Beads (Smart-Lifesciences Biotechnology, Changzhou, China).

### 2.17. Statistical Analysis

For the statistical analysis, the data distribution was assumed to be normal. Data are presented as means ± standard deviation (SD). Statistical differences were assessed with Student’s *t*-test for two groups and one-way ANOVA for multiple groups, followed by Fisher’s least significant difference test. Statistics were generated using GraphPad Prism 7.0 software. A *p* value < 0.05 was considered statistically significant.

## 3. Results

### 3.1. Giardia-Induced Cell Death and Mitochondrial Damage in Macrophages

**Figure 1:**
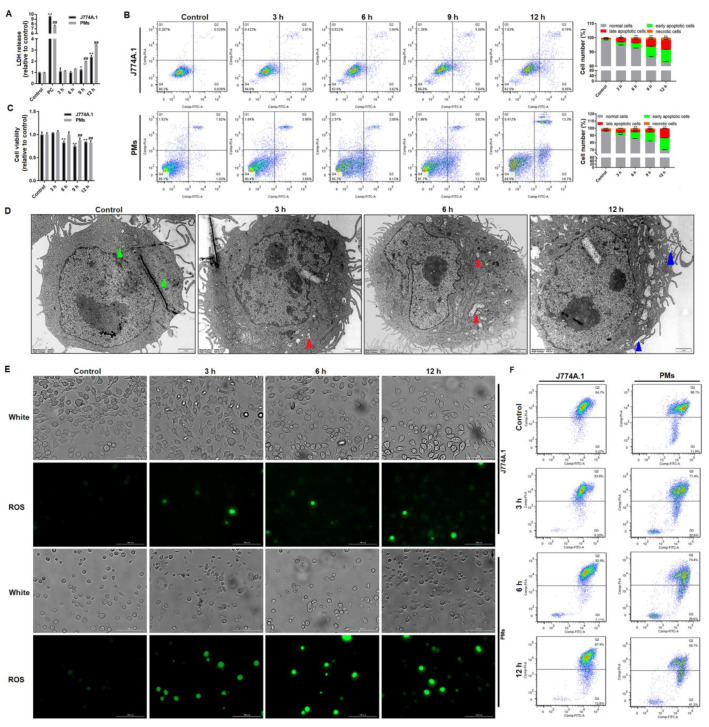
The negative effects of *Giardia* treatment on macrophages. J774A.1 cells or PMs were treated with trophozoites at a ratio of 10 parasites/cell for the indicated time periods, and then (**A**) LDH release in the culture supernatant was measured. (**B**) Annexin V-positive cells were analyzed with flow cytometry. Normal cells are in the lower left quadrant, necrotic cells in the upper left quadrant, early apoptotic cells in the lower right quadrant, and late apoptotic cells in the upper right quadrant. (**C**) Macrophage viability was examined by CCK-8 assay. (**D**) Damage of mitochondria and disruption of cell membrane integrity in PMs were observed by TEM (scale bar = 1 μm). Green arrows indicate normal mitochondria, red arrows indicate damaged mitochondria, and blue arrows indicate damaged plasma membrane. (**E**) ROS generation in macrophages was measured by fluorescence microscopy (scale bar = 100 μm). (**F**) The changes of MMP among groups were detected with flow cytometry. The horizontal axis gives the green fluorescence intensity, and vertical axis gives the red fluorescence intensity. All experiments were repeated at least three times. (**A**–**C**) Values are expressed as the mean ± SD (* *p* < 0.05, **, ^**##**^ *p* < 0.01 as compared to controls). (**D**–**F**) Representative pictures are shown.

We applied *Giardia* trophozoites to interact with J774A.1 cells and PMs of C57BL/6 mice to create in vitro infection models. Upon trophozoite treatment, LDH release was enhanced in a time-dependent manner in the culture supernatants, especially at 9 h and 12 h (*p* < 0.05), indicating cell membrane destruction (Figure 1A). LDH present in the extracellular media has been regarded as a reliable metric of pyroptosis-induced cytotoxicity [34]. When exposed to trophozoites, the population of Annexin V-positive J774A.1 cells and PMs increased in a time-dependent manner (*p* < 0.01, Figure 1B). The macrophage cell viability was significantly decreased after parasite incubation for >9 h compared with mock cells (*p* < 0.01, Figure 1C).

The ultrastructure of *Giardia*-treated PMs was examined by TEM. In *Giardia*-treated PMs, as indicated by the red arrows in Figure 1D, there was an increase in cristae disruption in some mitochondria. The link between mitochondrial damage and ROS overproduction is well known [35]. As illustrated in Figure 1E, trophozoite stimulation increased ROS production in J774A.1 cells and PMs in a time-dependent manner. In addition, trophozoite stimulation could also induce the MMP loss in macrophages, as indicated by a decrease in the red/green fluorescence intensity ratio (Figure 1F), which may be implicated in programmed cell death. And more notably, TEM micrographs showed that the plasma membrane integrity of PMs was disrupted at 12 h after treatment (blue arrows, Figure 1D), which is known as an important marker of pyroptosis [36].

### 3.2. Giardia-Induced NLRP3/CASP1/GSDMD-Mediated Macrophage Pyroptosis

**Figure 2:**
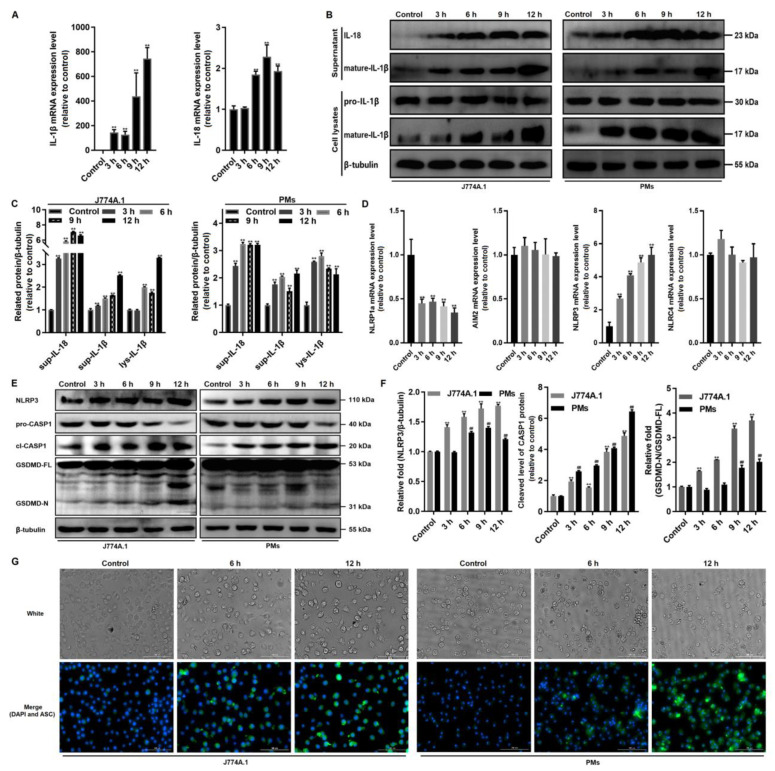
*Giardia*-induced NLRP3/CASP1/GSDMD-mediated macrophage pyroptosis. J774A.1 cells or PMs were treated with trophozoites at a ratio of 10 parasites/cell for the indicated time periods. (**A**) The mRNA levels of IL-1β and IL-18 in PMs were evaluated by qPCR. (**B**,**C**) The protein levels of mature-IL-1β and IL-18 were assessed by Western blot analysis. The abbreviation sup- stands for supernatant, and lys- for cell lysates. (**D**) The mRNA levels of inflammasome sensors in PMs were assessed by qPCR. (**E**,**F**) Representative Western blots and quantification of NLRP3, pro/cl-CASP1, and GSDMD-FL/N proteins are shown. The abbreviation GSDMD-FL stands for full-length GSDMD, and GSDMD-N for N-terminal GSDMD. (**G**) ASC protein expression was detected by immunofluorescence staining (scale bar = 100 μm). All experiments were repeated at least three times. (**A**,**C**,**D**,**F**) Values are expressed as the mean ± SD (**^,**##**^ *p* < 0.01 as compared to controls). (**B**,**E**,**G**) Representative pictures are shown.

In addition to an increase in LDH release due to plasma membrane rupture, CASP1-mediated activation and the release of IL-1β and IL-18 are also important indicators of pyroptosis [37,38]. We found that the mRNA expression of IL-1β and IL-18 was notably upregulated in *Giardia*-treated PMs (*p* < 0.01, Figure 2A). The Western blot analysis revealed that trophozoite stimulation could promote pro-IL-1β cleavage to its mature and biologically active form IL-1β, which is then released into the extracellular environment (*p* < 0.01, Figure 2B,C). Similarly, treated macrophages showed a strikingly increased production of IL-18 in the supernatant (*p* < 0.01, Figure 2B,C).

Proinflammatory cytokine release is mediated by inflammasome activation, which is essential for host defense against enteropathogens [39]. Four major inflammasome sensors, NLRP1a, AIM2, NLRP3, and NLRC4, were analyzed for their expression levels in treated and untreated cells, only NLRP3 expression was significantly upregulated at both the mRNA and protein levels (*p* < 0.01, Figure 2D–F). NLRP3 can initiate the canonical inflammasome assembly by recruiting pro-CASP1 with the ASC adaptor, which induces pyroptosis via cleavage and activation of CASP1 and GSDMD [17]. Our study noted *Giardia*-induced cleavage of CASP1 and the formation of N-GSDMD (Figure 2E,F); the latter would facilitate pyroptotic pore formation and IL-1β and IL-18 release. An increase in the fluorescence intensity of ASC was observed in treated cells (Figure 2G).

### 3.3. Giardia-Induced ROS/NLRP3/CASP1/GSDMD-Mediated Macrophage Pyroptosis

**Figure 3:**
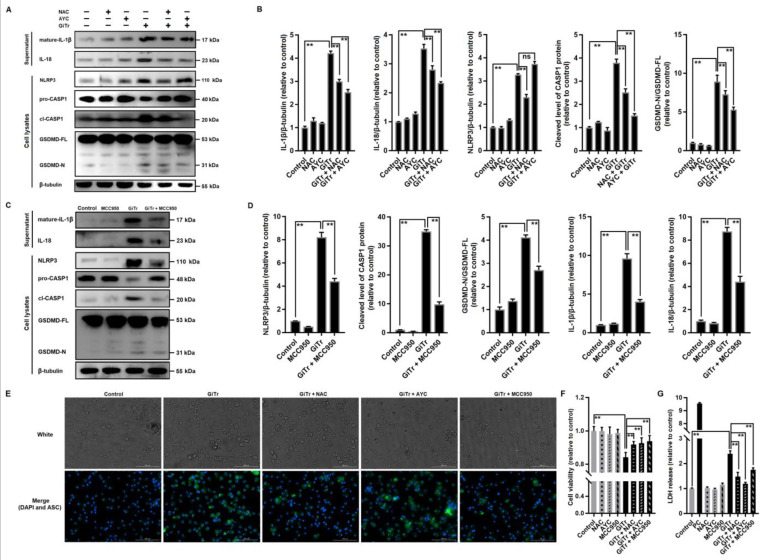
*Giardia*-induced ROS/NLRP3/CASP1/GSDMD-mediated macrophage pyroptosis. PMs were pretreated with 10 μM NAC, 40 μM AYC, or 50 μM MCC950 for 2 h, and stimulated with trophozoites at a ratio of 10 parasites/cell for 12 h. (**A**,**B**) The effects of NAC or AYC pretreatment on *Giardia*-induced upregulation of NLRP3, cleavage of CASP1 and GSDMD, and secretion of IL-1β and IL-18 were analyzed by Western blotting. (**C**,**D**) The effects of MCC950 pretreatment on *Giardia*-induced cleavage of CASP1 and GSDMD and secretion of IL-1β and IL-18 were analyzed by Western blotting. (**E**) After pretreatment with NAC, AYC, or MCC950, ASC expression in *Giardia*-treated PMs was detected by immunofluorescence assay (scale bar = 100 μm). (**F**,**G**) NAC, AYC, or MCC950 pretreatment affected the *Giardia*-induced decrease in cell viability and increase in LDH release. All experiments were repeated at least three times. (**A**,**C**,**E**) Representative pictures are shown. (**B**,**D**,**F**,**G**) Values are expressed as the mean ± SD (** *p* < 0.01 as compared to nontreated controls or as indicated). GiTr: reads *Giardia* trophozoites, and ns: no significant difference.

To identify the mediators leading to *Giardia* infection-associated GSDMD cleavage, we used N-acetylcysteine (NAC) and Ac-YVAD-CMK (AYC) to inhibit ROS generation and CASP1 activation, respectively. Pretreatment of PMs with NAC before exposure to trophozoites could inhibit the activation of NLRP3, cleavage of CASP1 and GSDMD, and the secretion of IL-1β and IL-18 (*p* < 0.01, Figure 3A,B). In comparison with NAC, AYC had similar inhibitory effects except that it could not block NLRP3 activation (Figure 3A,B). In addition, the suppression of NLRP3 by its inhibitor MCC950 can disturb the subsequent pyroptotic signaling (*p* < 0.01, Figure 3C,D). As visualized by immunofluorescence staining with an anti-ASC antibody, NAC and MCC950 pretreatment could suppress the upregulation of ASC expression in *Giardia*-stimulated PMs, while AYC did not exert a similar effect (Figure 3E). Taken together, the upstream regulator (ROS) and downstream effectors (ASC, CASP1, and GSDMD) of NLRP3 were determined. In addition, the reduced cell viability and increased LDH release in *Giardia*-treated PMs can both be alleviated in the presence of NAC, MCC950, or AYC (*p* < 0.01, Figure 3F,G).

### 3.4. Involvement of A20-Mediated NLRP3 Deubiquitination in Giardia-Induced Macrophage Pyroptosis

**Figure 4:**
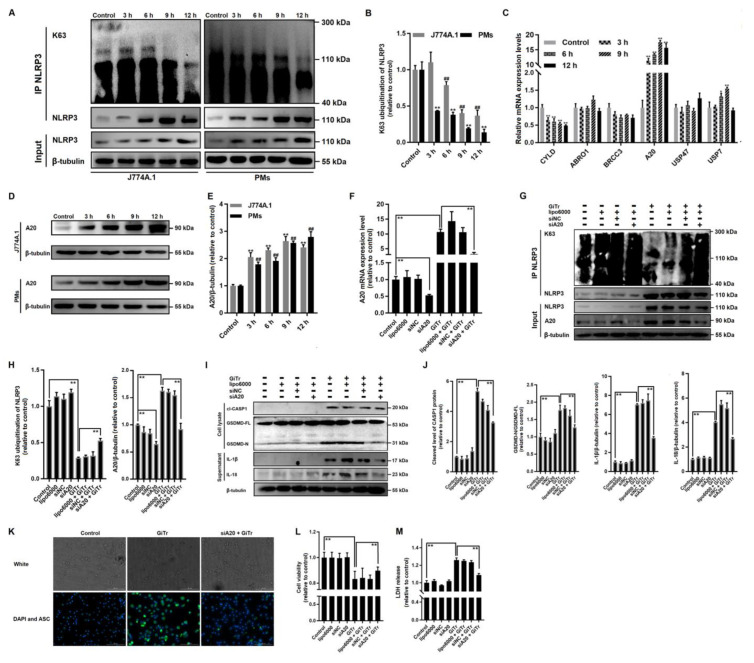
Involvement of A20-mediated NLRP3 deubiquitination in *Giardia*-induced macrophage pyroptosis. J774A.1 cells or PMs were treated with trophozoites at a ratio of 10 parasites/cell for the indicated time periods. (**A**,**B**) The levels of K63-linked ubiquitination of NLRP3 were assessed by co-IP analysis. (**C**) The mRNA expression levels of candidate DUBs in PMs were assessed by qPCR. (**D**,**E**) The protein level of A20 was analyzed by Western blotting. (**F**–**M**) PMs were transfected with siA20 or scrambled siRNA for 48 h and treated with trophozoites for 12 h. (**F**–**H**) The efficiency of A20 knockdown was detected by qPCR and Western blotting. (**G**,**H**) The effects of siA20 on NLRP3 deubiquitination and inflammasome activation induced by *Giardia* were analyzed by co-IP. (**I**,**J**) The effects of siA20 on *Giardia*-induced cleavage of CASP1 and GSDMD and IL-1β and IL-18 secretion were assessed by Western blotting. (**K**) Immunofluorescence analysis showed that siA20 affected ASC expression in *Giardia*-treated PMs (scale bar = 100 μm). (**L**,**M**) The effects of siA20 on decreased cell viability and enhanced LDH release induced by *Giardia*. All experiments were repeated at least three times. (**A**,**D**,**G**,**I**,**K**) Representative pictures are shown. (**B**,**C**,**E**,**F**,**H**,**J**,**L**,**M**) Values are expressed as the mean ± SD (* *p* < 0.05, **^,**##**^ *p* < 0.01 as compared to nontreated controls or as indicated). GiTr: reads *Giardia* trophozoites.

Emerging evidence demonstrates that NLRP3 inflammasome activation involves its deubiquitination [40]. It was indicated here in co-IP analysis that NLRP3 was decorated by K63-linked polyubiqutin chains, while trophozoite treatment significantly reduced the amounts of K63-linked polyubiquitin chains on NLRP3 (*p* < 0.01, Figure 4A,B). Protein ubiquitination can be reversed through the removal of ubiquitin from target proteins by deubiquitinating enzymes (DUBs) [41]. We examined the mRNA expression levels of six candidate DUBs (A20, CYLD, ABRO1, BRCC3, USP47, and USP7), only A20 expression exhibited a time-dependent increase in treated PMs (*p* < 0.01, Figure 4C). The upregulation of A20 expression was then confirmed by Western blot analysis (*p* < 0.01, Figure 4D,E). The siRNA-mediated knockdown of A20 was conducted to elucidate the potential role of A20 in regulating NLRP3 deubiquitination and the resulting pyroptosis. As predicted, the A20 mRNA and protein expression were effectively decreased by 48% (*p* < 0.01, Figure 4F) and 35% (*p* < 0.01, Figure 4G,H), respectively, after transfection with siA20. The enhanced A20 mRNA and protein expression in *Giardia*-treated PMs were inhibited by 70% (*p* < 0.01, Figure 4F) and 44% (*p* < 0.01, Figure 4G,H) by A20 knockdown, respectively. More importantly, the A20 knockdown could significantly alleviate *Giardia*-induced K63 deubiquitination of NLRP3 (*p* < 0.01, Figure 4G,H). In follow-up experiments, we found that *Giardia*-induced cleavage of CASP1 and GSDMD and the secretion of IL-1β and IL-18 could both be suppressed in A20-knockdown PMs (*p* < 0.01, Figure 4I,J). The A20 knockdown could also affect the expression of ASC in *Giardia*-treated PMs since a weaker fluorescence was observed in the group pretreated with siA20 than in the untreated group (Figure 4K). The A20 knockdown could also rescue the reduced cell viability induced by *Giardia* (*p* < 0.01, Figure 4L), as well as block *Giardia*-induced LDH release enhancement (*p* < 0.01, Figure 4M).

### 3.5. Involvement of TLR4 Activation in Giardia-Induced Macrophage Pyroptosis

**Figure 5:**
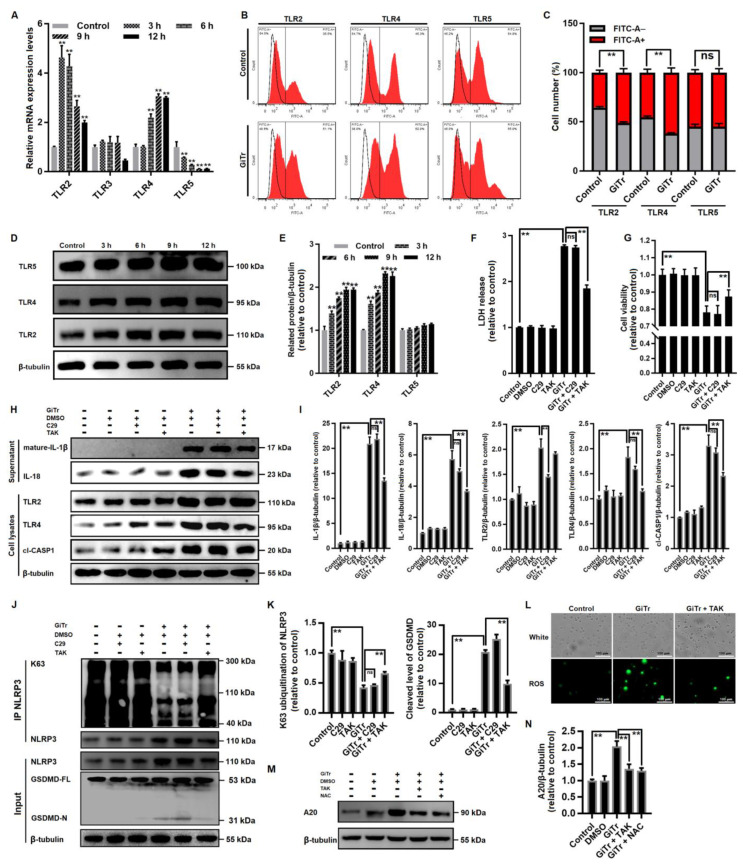
Involvement of TLR4 activation in *Giardia*-induced macrophage pyroptosis. PMs were treated with trophozoites at a ratio of 10 parasites/cell for the indicated time periods. (**A**) The mRNA levels of TLRs were assessed by qPCR. (**B**,**C**) The expression of TLRs on PM surfaces were detected by indirect immunofluorescence staining and flow cytometry. (**D**,**E**) The protein levels of TLR2 and TLR4 were assessed by Western blot analysis. (**F**–**N**) PMs were pretreated with 20 μM C29, 10 μM TAK, or 10 μM NAC for 2 h, and then stimulated with trophozoites for 12 h. (**F**,**G**) The cell viability and LDH release were detected. (**H**,**I**) The effects of C29 or TAK pretreatment on *Giardia*-induced CASP1 activation and IL-1β and IL-18 secretion were assessed by Western blotting. (**J**,**K**) The effects of C29 or TAK pretreatment on NLRP3 deubiquitination and GSDMD cleavage induced by *Giardia* were assessed by co-IP and western blotting. (**L**) Upon trophozoite treatment, ROS production in C29 or TAK-pretreated PMs was examined by fluorescence microscopy (scale bar = 100 μm). (**M,N**) The effect of TAK or NAC pretreatment on A20 upregulation induced by *Giardia* was analyzed by Western blotting. All experiments were repeated at least three times. (**A**–**C**,**E**–**G**,**I**,**K**,**N**) Values are expressed as the mean ± SD (** *p* < 0.01 as compared to nontreated controls or as indicated). (**D**,**H**,**J**,**L**,**M**) Representative pictures are shown. GiTr: reads *Giardia* trophozoites, and ns: no significant difference.

TLRs play vital roles in the innate reaction to microbial products and transmit specific immune responses against pathogens [22]. Here we evaluated the mRNA expression levels of TLR2, TLR3, TLR4, and TLR5 in *Giardia*-treated and untreated PMs (Figure 5A), among which only TLR2 and TLR4 showed a marked increase in expression (*p* < 0.01). Flow cytometry analysis indicated that TLR2 and TLR4 were strongly upregulated at 12 h post-treatment (*p* < 0.01, Figure 5B,C). Similar results to those seen in flow cytometry were also seen in Western blotting (*p* < 0.01, Figure 5D,E). The data imply that trophozoite stimulation activated TLR2 and TLR4 in PMs. We then used the TLR2 inhibitor TLR2-IN-C29 (C29) and the TLR4 inhibitor TAK-242 (TAK) to pretreat PMs to explore the potential association of TLR2 or TLR4 with *Giardia*-induced pyroptosis. TAK showed an inhibition of LDH release enhancement and recovered the reduced cell viability during *Giardia*-macrophage in vitro interactions (*p* < 0.01), while this was not the case for C29 (Figure 5F,G). *Giardia*-activated TLR2 and TLR4 can both be suppressed by their specific inhibitors (*p* < 0.01, Figure 5H,I); however, we found that only TLR4 inhibition by TAK could block CASP1 activation and IL-1β and IL-18 secretion (*p* < 0.01, Figure 5H,I). In addition, TAK also had obvious inhibitory effects on the K63 deubiquitination of NLRP3 and GSDMD cleavage (*p* < 0.01, Figure 5J,K). It is noteworthy that *Giardia*-induced ROS overproduction could be alleviated by TLR4 inhibition by TAK (Figure 5L), and pretreatment with TAK and NAC both suppressed the A20 upregulation in *Giardia*-treated PMs (*p* < 0.01, Figure 5M,N). Collectively, TLR4-mediated ROS production seems to regulate A20 expression and control pyroptotic outcomes in response to *Giardia* infection.

### 3.6. Giardia-Secreted PPIB Independently Induced Macrophage Pyroptosis

**Figure 6:**
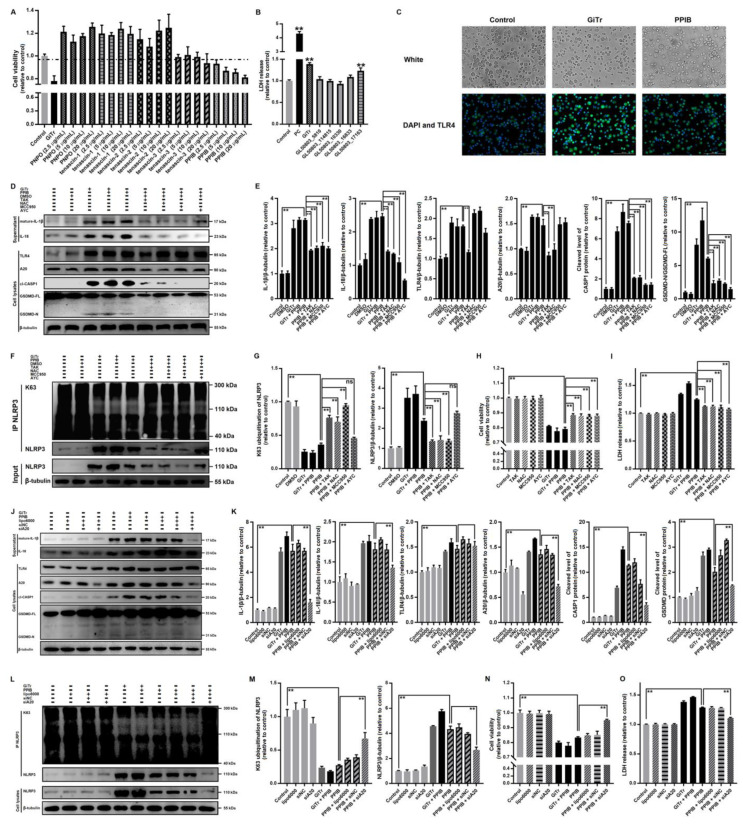
*Giardia*-secreted PPIB independently induced macrophage pyroptosis. PMs were treated for 12 h with *Giardia* trophozoites at a ratio of 10 parasites/cell and with the purified recombinant proteins at the indicated concentrations. (**A**) Cell viability among groups was assessed. (**C**–**O**) A protein concentration of 20 μg/mL was used for stimulation. (**B**) The levels of LDH release were assessed among groups. (**C**) TLR4 activation was assessed by immunofluorescence staining (scale bar = 100 μm). (**D**–**I**) The inhibitor TAK/NAC/MCC950/AYC was applied for 2 h before treatment. (**D**–**G**) Western blot and co-IP analyses indicated that PPIB triggered macrophage pyroptosis by a similar mechanism as parasites. (**H**,**I**) Cell viability and LDH release were affected by the same signaling. (**J**–**O**) The siA20 and scrambled siRNA were applied for 48 h before treatment. (**J**–**M**) Western blot and co-IP analyses indicated that K63 deubiquitination of NLRP3 by A20 was essential for PPIB/*Giardia*-induced macrophage pyroptosis. (**N**,**O**) Cell viability and LDH release were also affected by A20 inhibition. All experiments were repeated at least three times. (**A**,**B**,**E**,**G**–**I**,**K**,**M**–**O**) Values are expressed as the mean ± SD (** *p* < 0.01 as compared to nontreated controls or as indicated). (**C**,**D**,**F**,**J**,**L**) Representative pictures are shown. GiTr: reads *Giardia* trophozoites, and ns: no significant difference.

It can be concluded from the statements above that *Giardia* can induce macrophage pyroptosis via TLR4 signaling. Since noninvasive *Giardia* trophozoites cannot interact directly with macrophages in the gut lumen [5], we hypothesized that the secreted virulence factors probably function as a trigger to activate TLR4. As mentioned earlier, five secreted proteins PNPO, PPIB, and tenascin-1/2/3 are highly expressed in the culture supernatants of *Giardia* trophozoites [31,32]. We initially obtained five recombinant proteins of them using prokaryotic expression systems. The recombinant His6-tagged PNPO and PPIB were expressed as soluble proteins in *E. coli*, while the expressed tenascins were present in the form of inclusion bodies. After endotoxin removal if contaminated, these proteins were used to treat PMs, meanwhile, the *Giardia*-treated group was set as a positive control. We found that PPIB had a significantly negative effect on macrophage viability in a dose-dependent manner (*p* < 0.01, Figure 6A). PPIB stimulation could also induce LDH release from macrophages as was seen in trophozoite stimulation experiments (*p* < 0.01, Figure 6B). However, other tested proteins had no such effect (Figure 6A,B).

We then assessed the relevance of PPIB to the pyroptosis occurrence. PPIB also increased the protein levels of TLR4 similarly to those induced by *Giardia* (Figure 6C). The effects of PPIB stimulation on the expression of pyroptosis-related proteins are similar to those of trophozoite stimulation. For example, PPIB stimulation could promote CASP1 and GSDMD cleavage and IL-1β and IL-18 secretion in macrophages (*p* < 0.01, Figure 6D,E), and the related inhibitors of the upstream signal molecules including TAK, NAC, MCC950, or AYC could reverse these changes (*p* < 0.01, Figure 6D,E). In addition, PPIB-induced upregulation of A20 could be significantly repressed by TLR4 inhibition via TAK or by ROS inhibition via NAC, rather than by the inhibition of its downstream signaling molecule NLRP3 via MCC950 or CASP1 via AYC (Figure 6D,E). The co-IP assay revealed that PPIB stimulation induced K63 deubiquitination of NLRP3 and this process could be blocked by TAK, NAC, or MCC950 (*p* < 0.01, Figure 6F,G), while inhibition of CASP1 via AYC had no such blocking effects on its upstream molecule NLRP3. All the inhibitors just referred to could induce an increase in cell viability and a decrease in LDH release in PPIB-stimulated PMs (*p* < 0.01, Figure 6H,I).

The knockdown of A20 by a specific siRNA could significantly decrease the levels of cleaved CASP1 and GSDMD and extracellularly released IL-1β and IL-18 in PPIB-treated PMs, but had little effect on PPIB-activated TLR4 (Figure 6J,K). Importantly, A20 knockdown could suppress the activation of NLRP3 by modulating its deubiquitination (*p* < 0.01, Figure 6L,M), as well as reverse the decreased cell viability and increased LDH release induced by PPIB (*p* < 0.01, Figure 6N,O).

## 4. Discussion

Macrophages are primary effector cells of the innate immune system that form the first line of defense against microbial pathogens [42]. In this study, we found that *Giardia* and its secreted PPIB were able to induce macrophage pyroptosis and promote the release of the proinflammatory cytokines IL-1β and IL-18. Mechanistically, TLR4 was the first upstream molecule that was activated by PPIB or trophozoites, which triggered ROS overproduction and A20 upregulation. A20 promoted inflammasome activation by deubiquitinating NLRP3, driving pyroptotic cell rupture and IL-1β and IL-18 release. The resulting inflammatory reactions promote antimicrobial host defense as noted in [43].

*Giardia* trophozoites primarily colonize the proximal small intestine and do not invade host cells [44]. *Giardia*-secreted giardipain-1 and cysteine proteases have been identified as potent inducers of intestinal epithelial barrier breakdown [28,29], and the broken IEC barrier facilitates the process of PAMP (perhaps via certain secreted proteins) recognition by innate immune cells, notably macrophages that populate the intestinal lamina propria, to initiate immune responses required for the maintenance of intestinal homeostasis. Besides, *Giardia* trophozoite-secreted proteins like cathepsin B proteases and tenascins can disrupt the normal physiological function of IECs [31]. Peptidyl prolyl cis/trans isomerases (PPIases) are a superfamily of proteins ubiquitously distributed in both prokaryotes and eukaryotes [45]. Most characterized PPIases were previously considered to be involved in the folding and structuring of unfolded and partially folded polypeptide chains and proteins [46], whereas some were later identified as virulence-associated proteins upregulated during infection [47]. The roles of the highly variable PPIases in microbial pathogenesis remain little known [48]. Here *Giardia*-secreted PPIB was proved as a virulence protein that can trigger macrophage pyroptosis via TLR4 signaling. Actually, it has been reported that the secreted PPIase of *Helicobacter pylori*, HP0175, is able to induce apoptosis of the gastric epithelial cells in a TLR4-dependent manner [49], and it can also drive Th17 inflammation in gastric adenocarcinoma as described in [50]. Yet, the detailed role of PPIB in giardiasis remains to be explored.

Innate immune cells have complex signaling pathways for sensing pathogens, among which the most significant one is the TLR-mediated recognition of extracellular stimuli [51]. TLRs function as the effectors of immune surveillance and induce the transcriptional activation of proinflammatory factors [52]. It was reported that *Giardia* infection can enhance the production of proinflammatory cytokines, such as TNF-α, IFN-γ, IL-6, and IL-12 p40 in PMs, through the TLR2-mediated activation of MAPK signaling [13]. A recent study found that *Giardia* EVs trigger IL-1β, IL-6, and TNF-α secretion from mouse PMs via promoting the nuclear translocation of the NF-κB p65 subunit [53]. Another recent study identified TLR2 as a mediator for pyroptotic signaling in mouse PMs induced by *Giardia* EVs [25]. In contrast, our study revealed a significant increase of both TLR2 and TLR4 expression in *Giardia*-treated macrophages. We further found that TLR4 inhibition exerted a significant effect on impeding *Giardia*-induced NLRP3 activation and macrophage pyroptosis, while this is not the case for TLR2 inhibition. It is of interest to note that in macrophages NLRP3 can be activated by up to a 4-h stimulation with LPS and then ATP, while a prolonged (12 to 24 h) exposure dampens NLRP3 activation [54]. By contrast, our study showed that *Giardia* and its secreted PPIB could trigger the upregulation of NLPR3 in macrophages within 12 h of treatment; however, more data are needed to determine if any changes occur when subjected to a prolonged stimulation. We also noted a relatively high level of TLR5 expression in a small population of *Giardia*-treated macrophages, which might be attributed to the stimulus trophozoite flagellin [55]. The pyroptosis executioner GSDMD is known as a substrate for CASP1/4/5/11 [17]. Our study showed that *Giardia* was able to activate CASP1 to promote the cleavage of GSDMD. It has been demonstrated that CASP3-dependent cleavage of GSDME could be induced by chemotherapy [56], while no obvious cleavage of CASP3 was observed here. CASP1 is the best-described inflammatory caspase that requires inflammasome sensors like NLRP1a, AIM2, NLRP3, or NLRC4 for its activation [21]. Of the candidate sensors tested in this study, NLRP3 was confirmed to be active. *Neospora caninum* infection has also been shown to initiate NLRP3 inflammasome activation [57]. NLRP3 inflammasome activation is also present in macrophages affected by some other protozoan parasites, such as *Plasmodium*, *Leishmania*, *Trypanosoma*, *Toxoplasma*, and *Entamoeba* [20]. The NLRP3 inflammasome pathway contributes to host defense against infectious pathogens due to subsequent IL-1β and IL-18 secretion. IL-1β is a potent proinflammatory mediator implicated in recruiting immune cells to the site of infection and stimulating adaptive immune responses, and IL-18 induces the sequential activation of natural killer cells and cytotoxic T cells [58]. Mouse macrophages seem dispensable for anti-*Giardia* protective immunity [59], yet they could at least initiate an inflammatory response as reflected here.

NLRP3-mediated macrophage pyroptosis is associated with various kinds of infections, but the mechanisms of activation are complex [20]. Post-translational modifications of proteins such as ubiquitination are important for many biological processes [60]. NLRP3 is ubiquitinated with K63- and K48-linked polyubiquitin chains [61], among which the K63-linked polyubiquitin chain presumably serves as a molecular platform for various signaling pathways. Protein ubiquitination can be reversed by DUBs as indicated earlier. It has been known that BRCC3, in combination with ABRO1, can form a cytosolic BRISC complex and modulate K63-linked ubiquitination of NLRP3 [40]. Yet here we found that *Giardia* trophozoite stimulation modified the ubiquitination status of NLRP3 by upregulation of A20. The A20-promoted K63 deubiquitination of TRAF6 in macrophages induced by intracellular *Leishmania* leads to a remarkable reduction in TLR2-mediated IL-12 and TNF-α production [62]. In the present study, we confirmed the involvement of A20-mediated NLRP3 deubiquitination in inflammasome activation, CASP1 and GSDMD cleavage, and IL-1β and IL-18 release during *Giardia* infection. ROS production has been widely described as one of the earliest cellular responses following pathogen infection [63]. Interestingly, here we confirmed the positive relation between TLR4-induced ROS production and A20-mediated NLRP3 deubiquitination. In addition, it is also worth identifying the signaling mechanisms that affect *Giardia*-induced ROS production and inflammasome activation in macrophages, although this was not seen in this study. Actually, a recent study has demonstrated that LPS-induced macrophage ROS accumulation is associated with TLR4-dependent MYD88/PI3K signaling [64].

Bone marrow-derived macrophages, resident or induced PMs, or macrophages from the spleen, liver, and lung isolated from mice are usually variable in their cytokine production, migratory capacity, and antimicrobial activity [65]. Our study investigated the murine macrophage cell line J774A.1 and thioglycollate broth-induced mouse PMs for their potential to produce an anti-*Giardia* pyroptotic inflammatory response. Although sound, the current findings need to be confirmed further by using intestinal resident macrophages. Intestinal macrophages are essential players in the regulation of immune homeostasis and host defense against *Giardia* in the gut [14,59]. While macrophage pyroptosis has been widely identified as an important innate immune effector mechanism against intracellular pathogens, this is not the case for *Giardia*, widely known as a kind of extracellular parasite. We indicated here the detailed recognition and response mechanisms required for *Giardia*–macrophage interactions and the occurrence of pyroptosis, and determined PPIB as the potential trigger that implemented this process. The findings lead the way to a better understanding of the host defense mechanisms against *Giardia*.

## Data Availability

The data presented in the current study are available upon request to the corresponding author.

## Supplementary Materials

The following are available online at https://www.mdpi.com/article/10.3390/cells10123425/s1, Table S1: Primer pairs used in qPCR analysis, Table S2: Primer pairs used for plasmid construction.
