# Supplementary material for: Giardia duodenalis and Its Secreted PPIB Trigger Inflammasome Activation and Pyroptosis in Macrophages through TLR4-Induced ROS Signaling and A20-Mediated NLRP3 Deubiquitination
Source: Cells. 2021 Dec 6;10(12):3425. doi: 10.3390/cells10123425 (PMC8700504; doi:10.3390/cells10123425)
Supplement: Supplementary file 1 [file cells-10-03425-s001.zip › cells-10-03425-s001/Table S1.pdf]

Table S1 Primer pairs used in qPCR analysis.

Gene	Accession no.	Primer (5' to 3')	Product size
IL-1β	XM_006498795.5	F: GCTGCTTCCAAACCTTTGAC<br>R: AGCTTCTCCACAGCCACAAT	121 bp
IL-18	XM_036154617.1	F: GACTCTTGCGTCAACTTCAAGG<br>R: CAGGCTGTCTTTTGTCAACGA	169 bp
NLRP1a	XM_036156459.1	F: CCACTGAGCTACTATGCAGTACA<br>R: ACAACATCTTCACACCACCATC	202 bp
AIM2	XM_036166274.1	F: GTCACCAGTTCCTCAGTTGTG<br>R: CACCTCCATTGTCCCTGTTTTAT	328 bp
NLRP3	XM_036156549.1	F: ATTACCCGCCCGAGAAAGG<br>R: CATGAGTGTGGCTAGATCCAAG	83 bp
NLRC4	XM_006524347.5	F: ATCGTCATCACCGTGTGGAG<br>R: GCCAGACTCGCCTTCAATCA	86 bp
BRCC3	NM_001358736.1	F: TACACTGGAACGGAAATGCG<br>R: ACGGTCCTTTCTCTTGTCAGA	108 bp
CYLD	XM_036154329.1	F: ACCCTACTGGGAAGAACGGAT<br>R: CGGTCTTGGATGTACTGTCCTAT	120 bp
ABRO1	NM_198017.3	F: CACGAAGGATTTTTGCTGGGA<br>R: TGAACAAGGCTGATGGTTATGG	120 bp
A20	XM_006512702.4	F: ACCATGCACCGATACACGC<br>R: AGCCACGAGCTTCCTGACT	159 bp
USP47	XM_006508273.5	F: GATGTGATTCCCTTGGATTGCT<br>R: AACCCCATTGGTGTATCTTCTTC	101 bp
USP7	XM_006522141.4	F: CCACCAAGAATTACTCAGAACCC<br>R: AAGGACCGACTCACTCAGTCT	337 bp
β-actin	NM_007393.5	F: AGTGTGACGTTGACATCCG<br>R: GCAGCTCAGTAACAGTCCGC	298 bp
