# Supplementary material for: Giardia duodenalis and Its Secreted PPIB Trigger Inflammasome Activation and Pyroptosis in Macrophages through TLR4-Induced ROS Signaling and A20-Mediated NLRP3 Deubiquitination
Source: Cells. 2021 Dec 6;10(12):3425. doi: 10.3390/cells10123425 (PMC8700504; doi:10.3390/cells10123425)
Supplement: Supplementary file 1 [file cells-10-03425-s001.zip › cells-10-03425-s001/Table S2.pdf]

Table S2 Primer pairs used for plasmid construction.

Gene (identity)	Primer (5’ to 3’)[a]
PNPO (GL50803_5810)	F: CCGCTCGAGATGGTGGATCCTAAGCTTATC
	R: CCGGAATTCTCAATTCTTGATGCGCCAGG
Tenascin (GL50803_114815)	F: CCGCTCGAGATGTGGTGCGGGATTTCG
	R: CCGGAATTCTCAATCGCTATAGAAACAGGC
Tenascin (GL50803_10330)	F: CCGCTCGAGATGCTTTTCGTTTCTCTCGC
	R: CCGGAATTCCTAGTGCTCGTGCTTGATTTC
Tenascin (GL50803_16833)	F: CCGCTCGAGATGCTCCTGCTTGCAGGTC
	R: CCGGAATTCCTAATATTCGCACTGCACACC
PPIB (GL50803_17163)	F: CCGCTCGAGATGAACTCTCCAGTTTCTGAC
	R: CCGGAATTCTTACTGGAGCACGCCACAGTC

[a] Unique restriction sites for XhoI and EcoRI were shown underlined.
